# Optimizing Staining Protocols for Laser Microdissection of Specific Cell Types from the Testis Including Carcinoma *In Situ*

**DOI:** 10.1371/journal.pone.0005536

**Published:** 2009-05-14

**Authors:** Si Brask Sonne, Marlene D. Dalgaard, John Erik Nielsen, Christina E. Hoei-Hansen, Ewa Rajpert-De Meyts, Lise Mette Gjerdrum, Henrik Leffers

**Affiliations:** 1 Department of Growth and Reproduction, Rigshospitalet, Copenhagen, Denmark; 2 Department of Pediatrics, Rigshospitalet, Copenhagen, Denmark; 3 Department of Pathology, Rigshospitalet, Copenhagen, Denmark; Katholieke Universiteit Leuven, Belgium

## Abstract

Microarray and RT-PCR based methods are important tools for analysis of gene expression; however, in tissues containing many different cells types, such as the testis, characterization of gene expression in specific cell types can be severely hampered by noise from other cells. The laser microdissection technology allows for enrichment of specific cell types. However, when the cells are not morphologically distinguishable, it is necessary to use a specific staining method for the target cells. In this study we have tested different fixatives, storage conditions for frozen sections and staining protocols, and present two staining protocols for frozen sections, one for fast and specific staining of fetal germ cells, testicular carcinoma *in situ* cells, and other cells with embryonic stem cell-like properties that express the alkaline phosphatase, and one for specific staining of lipid droplet-containing cells, which is useful for isolation of the androgen-producing Leydig cells. Both protocols retain a morphology that is compatible with laser microdissection and yield RNA of a quality suitable for PCR and microarray analysis.

## Introduction

Most tissues are composed of a variety of different cell types and therefore it is difficult to analyse gene expression and the biology of specific cell types based on DNA, RNA or protein preparations from the whole tissue [1]. This problem can be circumvented by using laser microdissection (LMD) [2], to prepare samples significantly enriched for specific cell types.

Adult testes are composed of seminiferous tubules containing germ cells in different stages of spermatogenesis, embedded in Sertoli cells, which act as nursing cells ensuring the correct environment for the germ cells. Peritubular myoid cells line the tubules, and androgen-producing Leydig cells are located between the tubules in the connective tissue together with other interstitial cells such as fibroblasts, lymphocytes, Leydig cell precursors, and blood and lymph vessels. Thus, a normal adult testis contains at least 25 distinct cell types and their relative abundance varies according to developmental stage [3], [4]. Since each cell type has a characteristic gene expression profile, it is impossible to investigate gene expression in specific cell types on RNA prepared from whole testis.

Carcinoma *in situ* of the testis (CIS), also known as intratubular germ cell neoplasia (ITGCN) is the precursor of the majority of testicular germ cell tumors (TGCTs) [5]. The CIS cells are usually found in the periphery of the seminiferous tubules embedded in Sertoli cells. Previous gene expression studies of CIS have been hampered by the fact that even in testes where 100% of the tubules are “CIS tubules”, CIS cells still only constitute about 5–10% of the cells in the testes [6] and thus, the RNA is markedly diluted. CIS is a symptom of the Testicular Dysgenesis Syndrome (TDS), which also comprises infertility, testicular maldescent and penile malformations [7]. TDS has been mimicked in several animal models [8]–[10], supporting the proposed origin of TDS in early fetal life, probably a disturbed hormonal milieu during early reproductive development causing a reduced testosterone action [11]. Microarray expression profiling of the specific cell types, especially the testosterone-producing Leydig cells, would give important clues to the mechanisms behind the disturbed development [12].

When microdissecting cells from frozen, dehydrated tissues, it can be very difficult to distinguish between the different cell types. The commonly used haematoxylin and eosin staining is not sufficient in testis tissue with many different cell types, therefore it is vital to have a specific staining protocol that can mark the cells for microdissection. For our studies of TDS, we needed to identify fetal germ cells, CIS cells and Leydig cells. For fetal germ cells and CIS cells we took advantage of their embryonic stem cell (ESC)-like properties [13], which includes expression of an alkaline phosphatase [14]–[17] whose activity can be detected by staining with Nitro-blue tetrazolium chloride and 5-bromo-4-chloro-3-indolyl phosphate (NBT BCIP). For steroid-producing Leydig cells we utilised the presence of lipid droplets in their cytoplasm and developed a staining method based on Oil Red O (ORO) that specificially stains lipid droplets.

## Materials and Methods

### Preparation of tissues and cell culturing

The collection and use of human tissue samples for this project was approved by The Regional Committee for Medical Research Ethics in Denmark (no. KF-01-186 and H-KF-012006-3472). A written informed consent from the patients to use the leftover tissues for research was obtained by an andrologist assisting with the semen cryopreservation or the attending urologist before the surgery. Small samples of human adult testicular tissues containing CIS or seminoma (a germ cell tumor composed of cells morphologically resembling CIS) were collected at the Department of Pathology, Rigshospitalet after careful examination by a pathologist. The rat samples were collected under conditions approved by the Danish Agency for Protection of Experimental Animals and by the inhouse Animal Welfare Committee of the Institute for Food and Veterinary Research, DTU.

Tissue samples from four seminomas were fixed overnight in formalin (4% w/v formaldehyde pH 7.0); Stieve's fixative (Solution I: 90 g HgCl_2_ in 1.5 L H_2_O; Solution II: 400 g 40% formaldehyde and 80 g 98% acetic acid, just before use mix 38 ml Solution I and 12 mL Solution II); or GR fixative (200 mL 37% Formaldehyde, 40 mL acetic acid added to 1 L of 0.05 M phosphate buffer, pH 7.4 (all fixatives were from VWR, Bie & Berntsen, Copenhagen, Denmark). After fixation the tissue samples were embedded in paraffin.

In addition, two rat and four human tissues were embedded in Tissue-Tech Optimal Cutting Temperature (OCT) compound (Sakura Fintek Europe, Zoeterwonde, NL) and rapidly frozen. The frozen tissues were cut (10 µm sections) and collected on either superfrost slides (for morphological examination) or nuclease and human nucleic acid-free membrane slides (Molecular Machines & Industries, Glatbrugg, Switzerland) (for evaluation of RNA integrity), and fixed immediately.

### RNA quality of frozen versus paraffin embedded tissue

Seminoma samples from four patients were used for evaluation of RNA integrity in frozen and paraffin embedded, formalin, Stieve's or GR fixed tissues. Two 10 µm sections were cut under RNAse free conditions and immediately transferred to lysis buffer (frozen sections) or stored under dry, dark conditions (paraffin embedded tissues) until RNA purification.

### Storage of cryosections

Cryosections were either stored at −80°C in a slide box, stored in absolute ethanol at −80°C, or the tissue was repeatedly removed from −80°C storage and sections cut from the same tissue samples after 0, 1, 3, 7 and 30 days. The experiment was performed in duplicates on two seminoma samples and two rat testes.

### Fixation of cryosections

We tested eleven different fixatives with regard to RNA quality, morphology and compatibility with the subsequent staining protocols, the fixatives were: 75% ethanol, 100% ethanol, 100% acetone, 100% methanol, 60% isopropanol, metacarn (6∶3∶1 methanol∶ chloroform∶acetic acid) modified metacarn (8∶1 methanol∶acetic acid), Carnoy's (6∶1∶3 ethanol∶acetic acid∶chloroform), Clarke's (3∶1 ethanol∶acetic acid), Meyer's (19∶1 ethanol∶acetic acid), and formalin. Fixation time was 10 min for all fixatives. The experiments were performed on two seminoma samples and two rat testes and the RNA evaluation was performed in duplicates.

### Staining protocols

#### The staining protocols were as follows

Mayer's haematoxylin (VWR, Bie & Berntsen): 10 sec incubation buffer [0.1 M TRIS pH 9.5; 0.1 M NaCl; 0.05 M MgCl_2_], 10 sec haematoxylin and 10 sec wash in diethylpyrocarbonate (DEPC) treated destilled water quickly followed by dehydration in ethanol (10 sec 62% ethanol, 2×10 sec 96% ethanol and 2×10 sec 100% ethanol).

Alkaline phosphatase with NBT BCIP [18]: 10 sec incubation buffer, 90–120 sec NBT BCIP solution [262.5 µg/mL *p*-Nitro-Blue tetrazolium chloride; 225 µg/mL 5-Bromo-4-chloro-3-indolyl phosphate dipotassium salt (both from Sigma-Aldrich, St Louis, MO, USA); 0.7% dimethyl formamide in relevation buffer], 10 sec in DEPC water, followed by dehydration in ethanol as described above.

Alkaline phosphatase with Kiel [19]: 2 min Kiel solution [6% ethylenglycol monoethylether (MERCK KGaA), 1 mM naphtol AS-BI phosphate (Sigma-Aldrich), 0.01% new fuchsin (VWR, Bie & Berntsen), 0.02% NaNO_2_ (VWR, Bie & Berntsen), 0.05 M TRIS-HCl buffer pH 8.7], 10 sec DEPC water and dehydration in ethanol as described above.

ORO [20]: 15 min ORO staining solution [0.3% Oil Red O (Sigma-Aldrich), 60% isopropanol], thorough wash in 60% isopropanol followed by DEPC water, 10 sec haematoxylin, 10 sec DEPC water and 10 sec dehydration in isopropanol.

RNA degradation after the different staining protocols was assessed in two seminoma samples that were sectioned and stained with haematoxylin, Kiel, NBT BCIP or ORO and two rat testes that were stained with haematoxylin and ORO. The RNA quality of sections from the four samples was examined immediately before and after the respective staining protocols and 4 h, 24 h and 72 hours after staining. All samples were run in duplicates.

### RNA purification and quality assessment

We used several different kits for testing the RNA quality of frozen versus paraffin embedded tissues. Frozen tissues: Nucleospin RNA II (Macherey Nagel, Düren, Germany) and Rneasy Mini Kit (Qiagen, Hilden, Germany); paraffin embedded tissues High Pure RNA Paraffin Kit (Roche, Basel, Switzerland), Absolutely RNA FFPE Kit (Stratagene, Cedar Creek, TX, USA), Pure Link FFPE RNA Isolation Kit (Invitrogen, Carlsbad, CA, USA) and Rneasy FFPE Kit (Qiagen). In addition both frozen and paraffin embedded tissues were purified using TRIzol reagent according to the manufacturer (Life Technologies, Gaithersburg, MD, USA), followed by precipitation with an equal amount of isopropanol, 0.1 volume 3 M sodium acetate (pH 5.2) and resuspension in RNAse free water. Furthermore, the samples were DNase treated using the RNAqueous-Micro Kit (Ambion, Austin, TX, USA) according to the provided protocol.

RNA from single frozen sections was purified using the Ambion RNAqueous micro kit (Ambion) according to the protocol provided by the manufacturer. The RNA quality was determined by the Bioanalyzer Picokit (Agilent Technologies).

The statistical significance was evaluated by Student's t-test. All analyses were two-tailed and a p value less than 0.01 and 0.05 was considered statistically significant.

## Results and Discussion

Isolation of RNA from specific cells by LMD is preceded by a series of steps starting already when the tissue is removed from the patient or animal. In principle, all manipulations including removal and collection of tissues, should be performed as fast as possible and the tissue samples should be kept on ice and transferred to −80°C as fast as possible, after thawing and sectioning the tissues should be in aqueous solution for as short time as possible as this is where the RNAses thrive and RNA quality decreases.

### Fixation and storage of tissue samples

**Figure 1:**
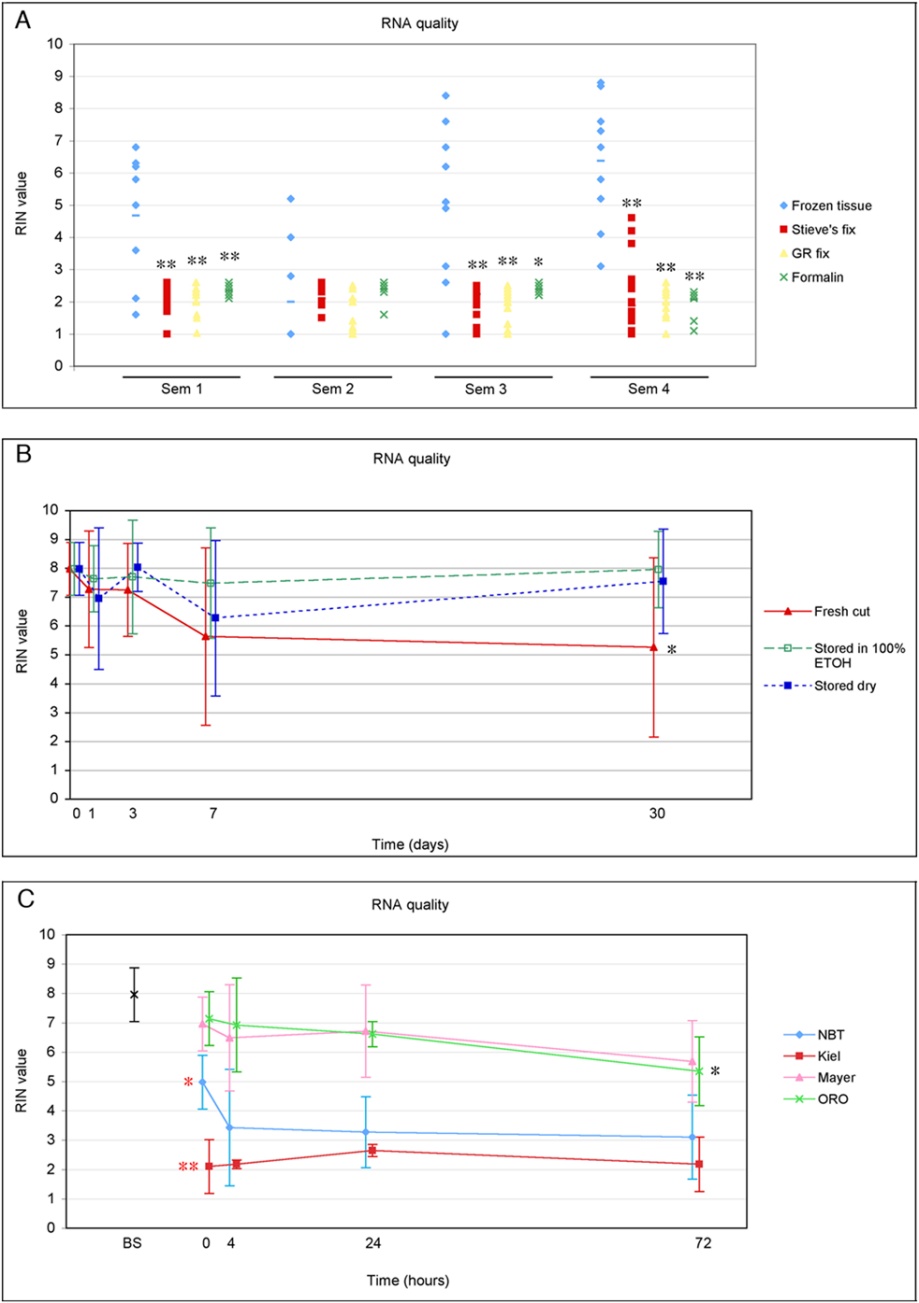
RNA quality of testicular tissues after different fixation protocols, storage and staining methods. The RNA quality was determined on an Agilent Bioanalyzer where higher RIN values reflect better RNA quality. Error bars show standard deviations. The statistical significance was evaluated by Student's t-test, * p<0.05; ** p<0.01. A: Frozen vs paraffin embedded tissues. Quality of RNA, purified from frozen sections (blue n = 9) or from tissues fixed in either formalin (green, n = 15), Stieves (red, n = 15) or GR fixative (yellow, n = 15) and embedded in paraffin. B: Storage of frozen sections. RNA quality in seminoma and rat testis after storage for 1, 3, 7 or 30 days as frozen sections at −80°C (blue, n = 8); storage at −80°C in 100% ethanol (green, n = 8); or sections freshly cut from a tissue sample that was repeatedly removed from −80°C (red, n = 8). C: Time-dependent RNA degradation after staining. Sections were fixed in 75% ethanol or 60% isopropanol, and stained according to the respective protocols (Tables 2 and 3). The RNA quality was assessed just before staining (BS) and 0, 4, 24 and 72 hours after staining. * and ** show that for the NBT BCIP and Kiel protocols, the RNA quality was significantly reduced already during staining (between BS and time 0) and * indicate significant differences during the subsequent storage (between 0 and 72 hours).

We tested the quality of RNA isolated from testicular tumor biopsies that were either frozen or fixed in different cross-linking fixatives (Stieve's, GR fixative and formalin) and embedded in paraffin (Fig. 1A).

Previous reports have shown that RNA-extraction from formalin fixed, paraffin embedded tissue results in small amounts of partially degraded and chemically modified RNA [21]–[23], although it may be sufficient to perform microarray analysis [24]–[25]. In our experience, the morphology of tissues fixed in Stieve's fixative and GR fixative is superior to the morphology of tissues fixed in formalin and they are excellent for *in situ* hybridisation (ISH) [26]. Therefore we tested if RNA extraction from Stieve's and GR fixed tissues could give a better RNA quality than formalin fixed tissues. RNA was purified using different commercial RNA purification kits, but we did not observe any systematic differences in RNA quality. In three of four seminoma samples, the frozen sections gave significantly better RNA quality than the fixed, paraffin embedded tissues, irrespective of the type of fixative used (Fig. 1A). Since a similar poor RNA quality was observed for paraffin embedded and frozen sections fixed in formalin, it is likely that it was the formalin fixation that caused the poor RNA quality, however wax embedding also negatively effects RNA quality [23]. There was a surprisingly high variation in the RNA quality derived from frozen sections (Fig. 1A), which may be caused by a higher susceptibility for RNA degradation in unfixed sections, but the time before transferral to lysis buffer is also critical. Frozen, ethanol-fixed tissues were later in the study used as controls and they showed a much more constant and high RNA quality. This verified that RNA from frozen tissue had the most optimal quality for subsequent microarray analysis.

When microdissecting single cells, it may be necessary to collect cells from >30 tissue sections. Depending on the pace of RNA degradation, the optimal storage of sections may become an important question. We had a suspicion that repeated thawing of frozen tissues resulted in degradation of the RNA, and were sceptic about storing the sections at −80°C under dry conditions. Therefore we tested different storage conditions for the cryosections: we either stored the ethanol-fixed and dried sections at −80°C in a slide box; transferred them to 100% ethanol at −80°C; or cut fresh sections from frozen tissues that were repeatedly thawed prior to sectioning. Repeated thawing of the tissue markedly compromised the morphology and the RNA integrity was significantly decreased (p<0.05) after 30 days (Fig. 1B). Freezing the sections at −80°C for 30 days in 100% ethanol or keeping them dry in a slidebox at −80°C did not affect the morphology, actually it seemed even better after storage in ethanol, and the RNA quality remained high after storage for one month (Fig. 1B).

The choice of fixative is based on obtaining the best possible morphology while preserving the RNA quality. We tested the morphology and the RNA quality of frozen sections after fixation in 11 different fixatives and at the same time tested the compatibility with our staining protocols (Table 1). Previous studies reported on optimal morphology and RNA quality using −20°C acetone, metacarn and 70% ethanol fixation [23], [27]–[28]. In our hands, fixation in 75% ethanol, 100% ethanol and acetone gave the best morphology (judged by haematoxylin staining), but other fixatives also resulted in an acceptable morphology (Table 1). However, the RNA quality, according to the RIN values (Bioanalyzer picokit), differed markedly between fixatives (Table 1). The best RNA quality was obtained after fixation in acetone, but most other fixatives also yielded good quality RNA. Not surprisingly, the worst RNA quality was seen after fixation in the crosslinking fixative formalin; although this gave a good morphology, and was compatible with all the staining protocols, the RNA was not suitable for the subsequent applications, with a RIN value below 2 (Table 1). Thus excluding formalin, sections can be cut from frozen tissue and fixed in a range of fixatives, that give a good morphology and still provide RNA of a good quality.

**Table 1:** Morphology, staining, and RNA quality after fixation in different fixatives.

Fixative	Morphology (Mayers haematoxylin)	ORO	NBT BCIP	Kiel	RIN (n = 8)
**75% Ethanol**	++/+++	−	++	−	6.9 (5.1–8.7)
**100% Ethanol**	+++	−	++	−	6.6 (4.6–8.5)
**Acetone**	+++	−	−	−	8.6 (8.0–9.2)
**Methanol**	++	−	+/−	−	7.4 (6.4–8.3)
**60% isopropanol**	++	+++	++	−	6.9 (6.5–7.4)
**Metacarn**	+/++	−	−	−	6.7 (5.3–8.1)
**Modified metacarn**	++	−	−	−	8.1 (7.4–8.7)
**Carnoys**	++	−	−	−	7.9 (7.3–8.6)
**Clarkes**	+	−	−	−	6.2 (4.3–8.1)
**Meiers**	+	−	−	−	7.1 (6.3–7.8)
**Formalin**	−/++	++	+/−	−	1.9 (1.0–2.8)

Morphological evaluation of haematoxylin stained tissue, compatibility with NBT BCIP, Kiel and ORO staining protocols and RNA quality of frozen tissues after 10 minutes fixation in different fixatives. The morphology was rated from haematoxylin stained tissues in a scale from + to +++, where + is poor morphology, ++ is intermediate and +++ is very good morphology. ORO, Kiel and NBT BCIP stainings were evaluated as absent (−), weak (+), medium (++) or strong (+++). The histological evaluations were performed by 2 independent observers. RNA quality was assessed using RIN values, average (95% confidence intervals).

### Staining of fixed tissue sections

We optimised short staining protocols using Kiel solution, NBT BCIP, ORO and haematoxylin. Because TGCTs and fetal germ cells retain the expression of the “ESC-specific” alkaline phosphatase enzyme visualized by NBT BCIP and Kiel, these staining protocols can be used to stain TGCTs [18], fetal gonocytes and oogonia [15], [16], and other tumours and ESC-like cells with ESC-like properties expressing alkaline phosphatase [14], [18]. NBT BCIP gave a strong dark blue colour after 90–120 sec in the staining solution and the staining was stable in ethanol. NBT BCIP staining gave the best result for sections fixed in 75% and 100% ethanol, and 60% isopropanol (Table 1), but sections fixed in methanol and formalin could also be stained (Table 1). None of the other tested fixatives were compatible with NBT BCIP staining. Kiel staining was excellent for microdissecting CIS cells for DNA preparations [29], however the staining was generally very weak after 2 min staining and initial experiments showed a very poor RNA quality after staining with Kiel for 5 min (results not shown). Thus, it is probably difficult to enhance the Kiel staining sufficiently to visualize CIS for LMD without damaging the RNA. NBT BCIP and Kiel staining resulted in a rapid decrease in RNA quality during staining (p<0.01 and p = 0.05 for Kiel and NBT BCIP, respectively) and a moderate degradation during storage (Fig. 1C).

**Figure 2:**
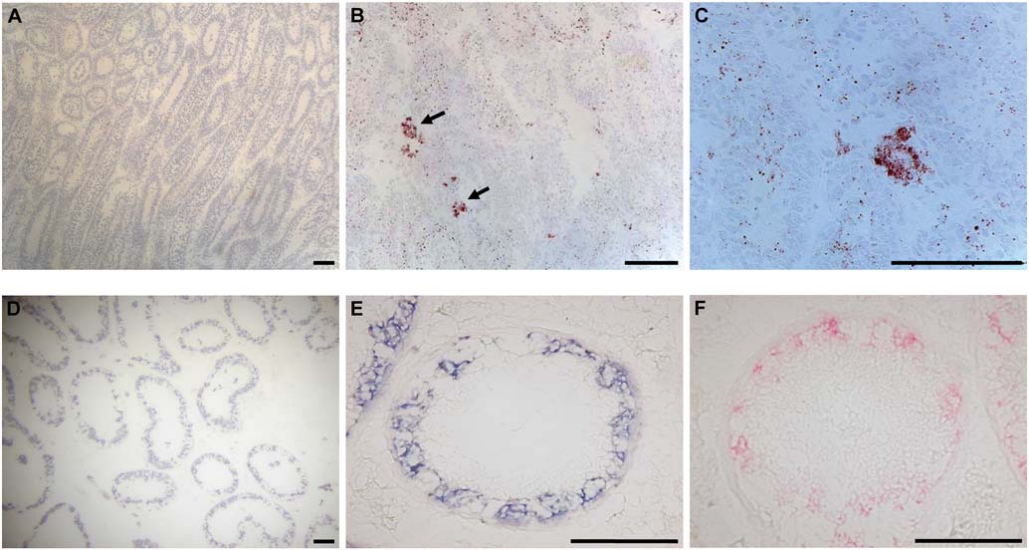
Examples of stained tissues. Top panel: Rat testes stained with haematoxylin (A) or haematoxylin and ORO (arrows) (B and C). Bottom panel: Carcinoma *in situ* stained with NBT BCIP for 120 sec (D and E) or Kiel for 5 min (F). Scale bars correspond to 100 µm.

ORO staining is dependent on the presence of lipid droplets in the cytoplasm and both fixation and staining therefore cannot include incubation in solvents that dissolve the droplets. Thus, ORO staining worked well on sections fixed in 60% isopropanol, but formalin fixed sections could also be stained (Table 1). ORO was excellent for staining a subset of Leydig cells in rat testes (Fig. 2), but the method can in principle be used for staining any cell with lipid droplets including adipocytes and steroid-producing cells in the adrenal gland. Finally, haematoxylin staining, which stains the nuclei in all tissues irrespective of fixative (Fig 2) was used for evaluation of the tissue integrity after fixation in 11 different fixatives (Table 1). In contrast to the NBT BCIP and Kiel staining protocols, ORO and Mayers both preserved the RNA quality and gave excellent RIN values even 72 hours after staining, however for ORO stained sections the RNA quality was significantly reduced after 72 hours (p<0.05), indicating that also for this staining it is advantageous to perform microdissection as soon as possible.

Previous studies have shown that it is possible to perform reliable gene expression profiling using partially degraded RNA [25], [30]–[31]. Accordingly, we have successfully performed microarray analysis on RNA from cells isolated by microdissection [32] according to the described staining protocols (Tables 2 and 3).

**Table 2:** NBT BCIP staining protocol.

10 min.	Fixation in 75% Ethanol
Up to 6 months	Optional: Storage at −80°C in 100% Ethanol
10 sec	Relevation buffer
90–120 sec	NBT BCIP solution
10 sec	DEPC H_2_O
10 sec	62% Ethanol
2×10 sec	96% Ethanol
2×10 sec	100% Ethanol
App. 5 min	Air dry

**Table 3:** ORO staining protocol.

10 min.	Fixation in 60% isopropanol
15 min	ORO solution
10–30 sec	Wash 60% isopropanol
10 sec	DEPC H_2_O
5 sec	Haematoxylin
10 sec	DEPC H_2_O
10 sec	Dehydration 100% isopropanol
App. 10 min	Air dry
